# Parallel changes of taxonomic interaction networks in lacustrine bacterial communities induced by a polymetallic perturbation

**DOI:** 10.1111/eva.12050

**Published:** 2013-02-18

**Authors:** Karine Laplante, Boutin Sébastien, Nicolas Derome

**Affiliations:** Département de Biologie, Institut de Biologie Intégrative et des Systèmes (IBIS), Université LavalQuébec, QC, Canada

**Keywords:** adaptation, bacterial communities, ecosystem services, next generation sequencing, polymetallic gradient contamination, taxonomical networks

## Abstract

Heavy metals released by anthropogenic activities such as mining trigger profound changes to bacterial communities. In this study we used 16S SSU rRNA gene high-throughput sequencing to characterize the impact of a polymetallic perturbation and other environmental parameters on taxonomic networks within five lacustrine bacterial communities from sites located near Rouyn-Noranda, Quebec, Canada. The results showed that community equilibrium was disturbed in terms of both diversity and structure. Moreover, heavy metals, especially cadmium combined with water acidity, induced parallel changes among sites via the selection of resistant OTUs (Operational Taxonomic Unit) and taxonomic dominance perturbations favoring the *Alphaproteobacteria*. Furthermore, under a similar selective pressure, covariation trends between phyla revealed conservation and parallelism within interphylum interactions. Our study sheds light on the importance of analyzing communities not only from a phylogenetic perspective but also including a quantitative approach to provide significant insights into the evolutionary forces that shape the dynamic of the taxonomic interaction networks in bacterial communities.

## Introduction

Anthropogenic activities such as mining and smelting trigger profound damage to aquatic ecosystems (van Dam et al. 2002; Eisler 2004). Rational rehabilitation strategies are an urgent requirement to help such ecosystems to recover their original functions. However, efforts to restore aquatic biomes frequently fail to fully re-establish ecosystem services (Peralta et al. 2010). Bacterial communities are integral service providers (Gutknecht et al. 2006), thus conservation and rehabilitation of an impacted area require a thorough understanding of how multiple interactive species adapt to face such dramatic environmental perturbations.

Metabolic plasticity is likely to underpin the fundamental contribution bacterioplankton make to aquatic ecosystems (Armitage et al. 2003; Comte and del Giorgio 2011). As such, environmental fluctuations elicit a rapid adaptive response at the community level. However, the processes underlying this metabolic plasticity remain unclear. There is some evidence that metabolic pathways of lacustrine bacterioplankton may simply be compartmentalized between the main phyla (*Actinobacteria*, *Betaproteobacteria*, *Alphaproteobacteria,* and *Bacteroidetes*) (Debroas et al. 2009). Such functional compartmentalization suggests that interacting networks of taxa may constrain the functional repertoire of the whole microbial community.

Where taxonomic functional compartmentalization exists, so does a suite of mechanisms to mitigate the impact of the loss of species richness from the microbial community. Horizontal gene transfer (HGT) is universally reported as the major mechanism to facilitate rapid adaptation in microbial communities (Shintani et al. 2008; Sobecky and Hazen 2009; Parnell et al. 2010; Zhaxybayeva and Doolittle 2011). The resultant fluidity of functional characteristics between taxa can potentially bypass keystone species within functional networks. However, HGT frequency and efficiency is known decrease with genetic divergence (Andam and Gogarten 2011) and this phenomenon would in turn limit community level adaptation where functional characteristics are discretely packaged among only distantly related taxa.

Moderate and transient environmental changes do not dramatically reduce the catalog of ecosystem services provided by bacterial communities even though metabolic function may be compartmentalized among phyla (de Bello et al. 2010). In fact, at intermediate levels of disturbance, diversity is maximized because both competitive K-selected and opportunistic R-selected species can coexist (Dial and Roughgarden 1988; Kassen et al. 2004). Alternative members of the community with similar functional characteristics, replace the lost species (Folke et al. 1996; Lee et al. 2010; Barberán et al. 2012). As such, a reticulate network of community-level functional interactions buffers moderate environmental perturbations (Laplante and Derome 2011). In contrast, strong and permanent environmental disturbances are expected to dramatically alter bacterial community diversity (Rohr et al. 2006; Deng et al. 2007; Parnell et al. 2009). Such perturbations, for example the presence of an industrial pollutant, are expected to erase many species either via the direct action of the pollutant, or – crucially - via secondary effects e.g. the loss of keystone species or phyla from the functional network.

Mining and smelting release toxic heavy metals through acid mine drainage (AMD). AMD severely impacted sites are extreme environments as very acidic, very low in organic matter and rich in heavy metals or metalloids. When in excess in living cells, trace metals can bind to physiologically sensitive intracellular molecules or organelles, leading to deleterious effect (Wallace et al. 2003). Polymetallic contamination restricts community membership to organisms well-adapted to strong acidity and elevated concentration of metal sulfides (Collon 2003; Shade and Handelsman 2011). In this respect, heavy metals exert a very strong selective pressure on bacterial communities, causing structural changes in the community itself and are usually accompanied with a reduction in the microbial biomass (Kandeler et al. 2000), and with a loss of biodiversity (Deng et al. 2007; Laplante and Derome 2011). Moreover, communities exposed to environmental stress like radionuclide or polymetallic contamination typically share more structural similarities when compared with communities from unpolluted lakes (Fields et al. 2005; Laplante and Derome 2011). Studying these patterns of convergent or parallel evolution provides an opportunity to identify key associated parameters (Schluter 2000; Derome and Bernatchez 2006; Derome et al. 2006). In a previous survey we detected clear evidence of parallel adaptation in a lake system of both connected and independent bacterioplankton communities using 16S-SSU-rDNA DGGE profiling (Laplante and Derome 2011). Initially, we showed that polymetallic contamination drove parallel adaptation at the taxonomic level in two independent bacterial communities. Furthermore, we also showed that long-term exposure to contaminant gradients drove significant taxonomic diversity changes within three interconnected bacterial communities (Laplante and Derome 2011). However, 16S-SSU-rDNA DGGE fingerprint techniques are insufficiently sensitive to allow quantification of species abundance and provide no access to the rare biosphere. In contrast, next generation amplicon deep sequencing of the 16S locus provides much greater sensitivity. Taxonomic diversity and taxon abundance can be simultaneously evaluated with high reproducibility (Pilloni et al. 2012). Such accurate microbial community profiles enable downstream network and systems analysis of interactions between taxa.

In the following experiment, barcoded 454-pyrosequencing was used to study the same lake system as in Laplante and Derome (2011), to provide deeper insight into (i) the membership and structural changes occurring in the bacterial communities under long-term exposure to polymetallic contaminants, (ii) the parallel changes occurring between two independent bacterial communities impacted by similar selective pressure and (iii) the related environmental process driving community changes in the studied system.

## Material and methods

### Study area and sampling sites

**Figure 1:**
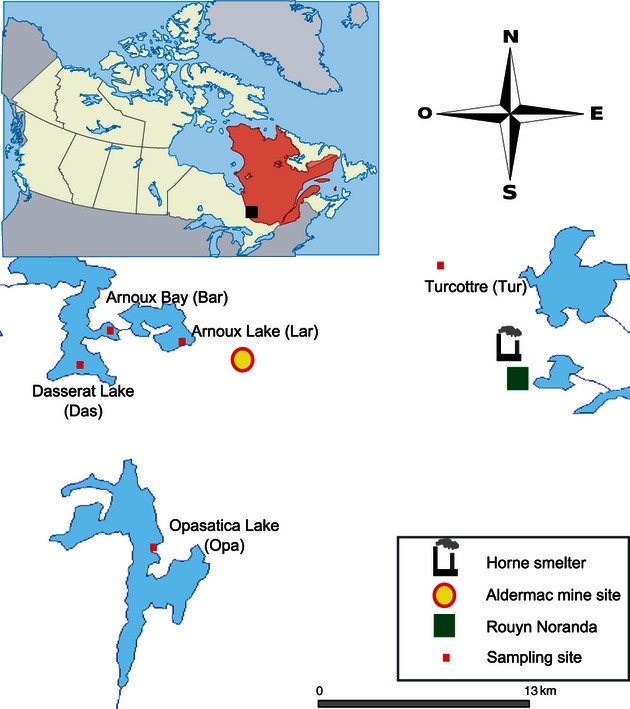
Geographical localization of Rouyn-Noranda (Abitibi-Temiscamingue, Canada) and the sampling sites visited in June 2010. Contaminated reference site: Turcotte Lake (Tur); clean reference site: Opasatica Lake (Opa); tested lake system: Arnoux Lake (Lar), Arnoux Bay (Bar), and Dasserat Lake (Das).

Detailed study area and sampling site information was detailed in Laplante and Derome (2011). Briefly, five sampling sites were chosen in the surroundings of Rouyn-Noranda (Québec, Canada) (see Fig. 1), where a strong mining activity generated polymetallic contaminant gradients (Couillard et al. 1993; Giguère et al. 2003, 2006). Opasatica Lake (Opa) was chosen as an unpolluted lake reference, while Turcotte Lake (Tur) was chosen as a contaminated reference. Hydrologically interconnected sites studied were Dasserat Lake (Das), Arnoux Bay (Bar) and Arnoux Lake (Lar). Through their interconnection, the water flow spreads the AMD from Arnoux Lake to Dasserat Lake such that a polymetallic gradient is naturally generated.

### Sampling and filtration procedures

These procedures were described in Laplante and Derome (2011). Briefly, sampling was done on June 2010 by collecting 6 L of water (3 L in duplicates) at 60 cm of depth in the water column. Water samples were filtered first with a 3.0 μm mesh size, followed by a 0.22 μm nitrocellulose membrane (Advantec) using a peristaltic filtration equipment (Masterflex L/S Pump System with Easy-Load II Pump Head; Cole-Parmer, Vernon Hills, IL, USA). Duplicates of the 0.22 μm membranes from the sampled lakes were placed into cryotubes containing 1 mL of sterile lysis buffer (40 mm EDTA, 50 mm Tris-HCl, 0.75 m sucrose) and then flash frozen into liquid nitrogen until DNA extraction.

### DNA extraction

Genomic DNA was extracted using a modified protocol of salt-extraction from Aljanabi and Martinez (1997). During the first lysis step, 113 μL of lysozyme (final concentration 1 mg/mL) was added to the sample and incubated for 45 min at 37°C. After this step, 113 μL of proteinase K (final concentration 0.2 mg/mL) and 450 μL of SDS 10% (final concentration 1%) were added to the lysate and incubated at 55°C over night with agitation. All the aqueous phase was transferred into a clean microcentrifuge tube containing 600 μL of 6m NaCl (final concentration 2.25 m), mixed and centrifuged 20 min at 17 000 *g*. The supernatant was transferred again into a clean microcentrifuge tube containing 1 volume of ice-cold isopropanol, mixed gently and stored 30 min at −20°C. The mixture was centrifuged 20 min at 16 162 g and the supernatant was thrown away. The pellet was washed with ice-cold 70% ethanol, air-dried and finally resuspended in 25 μL of sterile MilliQ H_2_O. Subsequently, DNA purity (260/280 ratio) and quantity were measured using a Nanodrop instrument (ND-1000, Nanodrop, Thermo Fisher Scientific, Waltham, MA, USA).

### Barcoded 16S pyrosequencing

Each sample, consisting of 12 ng of extracted genomic DNA, was PCR amplified using Takara Extaq Premix (Fisher). All PCR reactions were performed in a final volume of 50 μL containing 25 μL of Premix Taq, 1 μm of each primer and sterile MilliQ H_2_O to up to 50 μL. To achieve the PCR amplifications, a general reverse primer (R519) combined with B primer (Roche) was used in combination with a unique tagged forward primer (F63-targeted) combined with A primer (Roche) (see Table 1). PCR conditions were as follow: after a denaturing step of 30 s at 98°C, samples were processed through 30 cycles consisting of 10 s at 98°C, 30 s at 55°C, and 30 s at 72°C. The final extension step was done at 72°C for 4 min 30 s. Following amplification, samples were purified using AMPure Beads (Beckman Coulter Genomics, Denver, MA, USA). Samples were adjusted to 100 μL with EB (Qiagen, Hilden, Germany). Then, 63 μL of beads were added, the samples were mixed and incubated for 5 min at room temperature. Using a Magnetic Particle Concentrator (MPC), the beads were pelleted against the wall of the tube and the supernatants were removed. The beads were washed twice with 500 μL of 70% ethanol and incubated for 30 s each time. The supernatants were removed and the beads were allowed to air dry for 5 min. The tubes were removed from the MPC and 24 μL of EB were added. The samples were vortexed to resuspend the beads. Finally, using the MPC, the beads were pelleted against the wall once more and the supernatants were transferred to a new clean tube. The samples were quantified with Nanodrop and mixed equally then sent to the Plateforme d'Analyses Biomoléculaires (Institut de Biologie Intégrative et des Systèmes, Université Laval) to perform the pyrosequencing using the 454 GS-FLX DNA Sequencer with the Titanium Chemistry (Roche, Basel, Switzerland) using the procedure described by the manufacturer.

**Table 1:** Details of the primers used for the 16S barcoded pyrosequencing

Primer	Adaptor sequence	Barcode sequence	Primer sequence
R519	CCTATCCCCTGTGTGCCTTGGCAGTCT	–	CAGGWATTACCGCGGCKGCTG
F63_Lar	CCATCTCATCCCTGCGTGTCTCCGACTCAG	ACGAGTGCGT	CAGGCCTAACACATGCAAGTC
F63_Bar	CCATCTCATCCCTGCGTGTCTCCGACTCAG	ACGCTCGACA	CAGGCCTAACACATGCAAGTC
F63_Das	CCATCTCATCCCTGCGTGTCTCCGACTCAG	AGACGCACTC	CAGGCCTAACACATGCAAGTC
F63_Tur	CCATCTCATCCCTGCGTGTCTCCGACTCAG	AGCACTGTAG	CAGGCCTAACACATGCAAGTC
F63_Opa	CCATCTCATCCCTGCGTGTCTCCGACTCAG	ATCAGACACG	CAGGCCTAACACATGCAAGTC

Lar, Arnoux Lake; Bar, Arnoux Bay; Das, Dasserat Lake; Tur, Turcotte Lake; Opa, Opasatica Lake.

### Pyrosequencing data analysis

The open-source community-supported software program, Mothur (http://www.mothur.org) (Schloss et al. 2009), was used to process and analyze the sequence data following the Costello Stool Analysis tutorial (http://www.mothur.org/wiki/Costello_stool_analysis) (Costello et al. 2009). Following this tutorial, the filtration, the alignment and the trimming of the sequences was done to extract the high-quality reads at a uniform length of 300 nt to facilitate comparative analyses. Accordingly, the data were filtered using the following criteria: minimum average quality score: 35 for a windows of 50, number of differences to the primer sequence = 0, maximum number of differences to the barcode sequence = 0, number of ambiguous base calls = 0, maximium homopolymer length = 8. Then trimmed reads were grouped into clusters with a 97% identity threshold. The Costello Stool Analysis tutorial also provided the commands required to (i) calculate diversity and richness indexes (Nonparametric Shannon Index, Simpson Index, sequences coverage, abundance of unique OTUs, Chao1 estimate of richness), (ii) achieve the taxonomical survey of each community, (iii) build Venn diagrams at a cutoff of 3%, (iv) construct dendrograms showing either the taxonomical diversity similarities between communities (using the Jaccard Coefficient) or their taxonomical structure (i.e. OTU relative abundances) (using the Theta Coefficient) (Yue and Clayton 2005), and (v) determine whether the clustering within the trees is statistically significant using the Unifrac unweighted and weighted methods (Lozupone and Knight 2005).

### Statistical analysis

All data were first tested for normality and equality of variances (Zar 1999). Linear regressions were performed using the R command ‘lm(tableau$X∼tableau$Y)’ to test the existence of a linear relation between the response variable Y, namely the diversity indices and the taxonomical surveys of each community, and the explicative variable X, namely the abiotic data collected on the field (trace metals [Al, Cd, Cu, Fe, Mn, Pb, Zn], major cations [Ca, Mg, Na, K, S], DOC, pH and temperature). Then, analyses of variances (anovas) were undertaken using the R commands ‘model = lm(tableau$X∼tableau$Y)’ and ‘anova(model)’ to assess the factors explaining the variance of the models. Effects for the anova tests were deemed significant at *P* < 0.05 and marginally significant at *P* = 0.05−0.10. In addition, for each linear regression, the coefficient of determination (*R*^2^) was calculated to assess the validity of the model. The statistical analyses were performed using R statistical software (http:/http://www.r-project.org/).

### Bacterial network analysis

Based on the relative abundance of the 50 dominant species, the concentrations of cadmium, pH, and dissolved organic carbon (DOC) values measured in the five lakes, bacterial networks were built using Rcmdr, a platform-independent menu interface running with R software. The correlation coefficients were calculated by applying the Spearman algorithm. This previous filtering step removed poorly represented OTUs and reduced network complexity. We considered a valid co-occurrence event to be a robust correlation if the Spearman's correlation coefficient was both >0.6 and statistically significant (*P*-value <0.01) (Barberán et al. 2012). The networks were displayed using the Fruchterman-Reingold algorithm with 500 iterations. The nodes in the reconstructed networks represent the OTUs with 97% identity and the selected physicochemical parameters. Then, the edges (that is, connections) correspond to a strongly significant interaction between nodes and red edges indicate a negative interaction between two nodes.

## Results

### Environmental data

Trace metals (Al, Cd, Cu, Fe, Mn, Pb, Zn), major cations (Ca, Mg, Na, K, S), DOC, pH, and temperature for the studied lakes are summarized in Table 2. For more details about the abiotic survey procedure, see Laplante and Derome (2011).

**Table 2:** Abiotic parameters measured at each sampling site

	Detection limit	Lar	Bar	Das	Tur	Opa	Mean ± SD
Trace metals (mg/L)							
Fe	0.002	4.939	0.705	0.11	0.097	0.07	1.184 ± 2.116
Al	0.001	0.930	0.405	0.052	0.113	0.074	0.315 ± 0.372
Zn	0.0007	0.5574	0.2703	0.0347	0.1063	<0.0007	0.1939 ± 0.2282
Mn	0.0001	0.4421	0.2598	0.0036	0.0368	0.0019	0.1488 ± 0.1960
CU	0.0005	0.0507	0.0243	0.0075	0.0141	0.0029	0.0199 ± 0.0190
Pb	0.003	0.003	<0.003	<0.003	<0.003	<0.003	0.003 ± 7.916 E-05
Cd	0.0002	0.0010	0.0007	0.0002	0.0009	<0.0002	0.0006 ± 0.0004
Major cations (mg/L)							
S	0.02	22.03	13.16	5.10	2.33	1.93	8.91 ± 8.61
Ca	0.02	9.92	8.26	7.34	2.00	7.97	7.10 ± 3.00
Mg	0.002	3.490	2.675	1.987	0.434	2.452	2.207 ± 1.131
Na	0.01	1.26	1.24	1.21	0.68	3.24	1.53 ± 0.99
K	0.002	0.597	0.461	0.497	0.139	0.917	0.522 ± 0.280
Others							
Doc	0.5	4.4	1.8	7.2	3.8	7.7	5.0 ± 2.5
pH	0.05	3.77	4.69	7.11	4.91	7.64	5.62 ± 1.67
Temp.	0.5	19.0	17.5	17.0	17.0	16.5	17.4 ± 1.0

Lar, Arnoux Lake; Bar, Arnoux Bay; Das, Dasserat Lake; Tur, Turcotte Lake; Opa, Opasatica Lake; SD, Standard Deviation; Al, Aluminum; Ca, Calcium; Cd, Cadmium; Cu, Copper; Fe, Iron; K, Potassium; Mg, Magnesium; Mn, Manganese; Na, Sodium; Pb, Lead; S, Sulfur; Zn, Zinc; DOC, Dissolved Organic Carbon; Temp., Temperature.

### 454 data analyses

After trimming the primer sequences, barcodes and adapter tags, the average sequence length was 425 nt and the total dataset comprised 47 124 high quality sequences with a minimum length of 300 nt. Then, after removing the chimeras and retrieving the unique sequences, the analyses using Mothur generated multiple outputs, which are summarized in Table 3. The Nonparametric Shannon Index, which takes into account the fact that possible rare species were not collected during the sampling, was the highest in Opa (*H'* = 4.564), followed by Das (*H'* = 3.834), Tur (*H'* = 2.924), and Lar (*H'* = 2.161), while the lowest Shannon value was calculated for Bar (*H'* = 0.728). The estimated species richness, as seen with the Chao values, was the highest in Das (*S*_Chao_ = 2674.010), followed by Lar (*S*_Chao_ = 1794.754), Opa (*S*_Chao_ = 1308.438), and Tur (*S*_Chao_ = 1204.238), while the lowest richness was observed in Bar (*S*_Chao_ = 836.324).

**Table 3:** Data collected for each community using Mothur

Site	*N*_seqs_	Coverage (%)	Shannon	InvSimpson	*S*_obs_	*S*_Chao_
Lar	12 026	96.7	2.161	0.713	532	1794.75
Bar	9819	98.0	0.728	0.141	280	836.32
Das	8503	92.8	3.834	0.896	847	2674.01
Tur	5898	94.7	2.924	0.805	444	1204.24
Opa	1721	82.9	4.564	0.936	405	1308.44

Lar, Arnoux Lake; Bar, Arnoux Bay; Das, Dasserat Lake; Tur, Turcotte Lake; Opa, Opasatica Lake; *N*_seqs_ Number of Sequences; Shannon, Nonparametric Shannon Index; InvSimpson, Inversed Simpson Index; *S*_obs_, Number of OTUs observed and *S*_Chao_, Richness index.

### Relationship between lacustrine microbial communities

**Figure 2:**
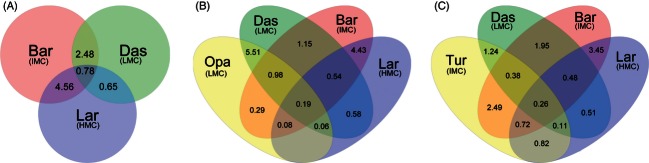
Venn diagrams of the percentage of species shared between communities at distance 0.03. The Venn diagrams were obtain using Mothur and allowed to primarily assess the percentage of species shared within (A) the tested lake system consisting in Arnoux Lake, high metallic contaminated communities group (Lar, HMC), Arnoux Bay intermediate metallic contaminated communities group (Bar, IMC) and Dasserat Lake, low metallic contaminated communities group (Das, LMC), and secondly to assess the percentage of species shared between the tested lake system and (B) the clean reference site Opasatica Lake (Opa, LMC) or (C) the contaminated reference site Turcotte Lake (Tur, IMC).

Venn diagrams of the percentages of species shared between communities (see Fig. 2) reveal that 4.56% of the OTUs were common between Lar and Bar, 2.48% were common to Bar and Das, 0.65% were shared between Das and Lar, while 0.78% of the OTUs were common to these three interconnected sites (see Fig. 2A). By analyzing the interconnected sites system with the reference communities (see Fig. 2B and C), we found that the unpolluted lakes Das and Opa exclusively shared the highest number of OTUs with 5.51%, followed by the moderately disturbed site Bar and the highly contaminated site Lar with 4.43% and 3.45% of exclusively shared OTUs when compared with either Opa or Tur, respectively. In contrast, Lar and Opa showed the lowest percentage of exclusively shared OTUs with 0%.

**Figure 3:**
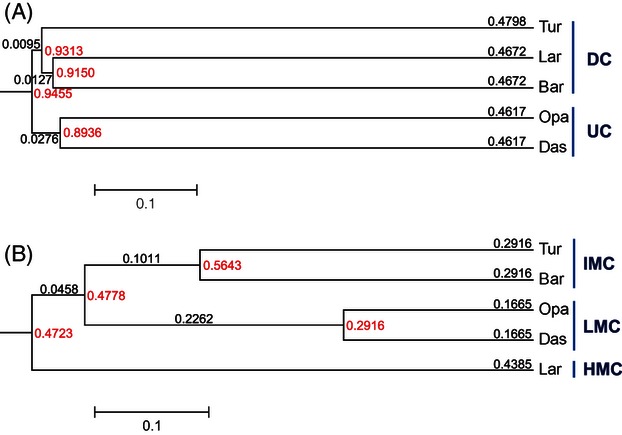
UPGMA dendrograms representing the membership and the composition shared between communities. The UPGMA dendrograms allowed to assess a) the membership (taxonomic diversity) shared between communities based on Jaccard coefficient (jclass) and b) the composition (taxonomic structure) shared between communities based on Yue & Clayton theta coefficient (thetayc). Branch lengths are displayed in black and values at the nodes are displayed in red. In (A), the communities clustered into two groups, either the disturbed communities (DC) group or the undisturbed communities (UC) group. In (B), following the polymetallic contamination magnitude over the taxonomic structure, the communities clustered into three groups, either the low metallic contaminated (LMC) communities group, the intermediate metallic contaminated (IMC) communities group or the high metallic contaminated (HMC) communities group. Contaminated reference site: Turcotte Lake (Tur); clean reference site: Opasatica Lake (Opa); tested lake system: Arnoux Lake (Lar), Arnoux Bay (Bar) and Dasserat Lake (Das). The scale bar represents 0.1 substitutions per alignment position.

The membership and structural similarities between the studied communities are illustrated by the distinct dendrograms provided using Mothur (see Fig. 3). Firstly, the membership dendrogram (see Fig. 3A), obtained from the unweighted (equally weighted) similarity matrix, showed two differentiated clusters with a Unifrac value at the node of 0.9455. The first cluster clearly regrouped the disturbed communities (DC) of Tur, Lar, and Bar (value at the node of 0.9313 and 0.0095 substitutions per alignment positions), while the second cluster strongly identified the undisturbed communities (UC) of Das and Opa (value at the node of 0.8936 and 0.0276 substitutions per alignment positions). Secondly, by evaluating the abundance of the different taxa, the weighted structural dendrogram (see Fig. 3B) indicated three differentiated clusters consistent with the magnitude of the contamination. The first cluster was the highly metallic contaminated (HMC) community of Lar (see Table 2 for detailed abiotic survey), which showed a significant unique taxonomical structure (value at the node of 0.4723 and 0.4385 substitutions per alignment positions). The other communities were grouped together under a similar cluster (value at the node of 0.4778 and 0.0458 substitutions per alignment positions) which comprised Tur and Bar under a subcluster, thus being identified as intermediate metallic contaminated (IMC) communities (value at the node of 0.5643 and 0.1011 substitutions per alignment positions from each other), and the low metallic contaminated (LMC) communities of Opa and Das under a highly significant subcluster (value at the node of 0.2916 and 0.2262 substitutions per alignment positions from each other). Evident from the nodes of both dendrograms, and despite the geographical connections between Das, Lar, and Bar, the Das community is more similar to the independent and undisturbed reference community at Opa.

**Figure 4:**
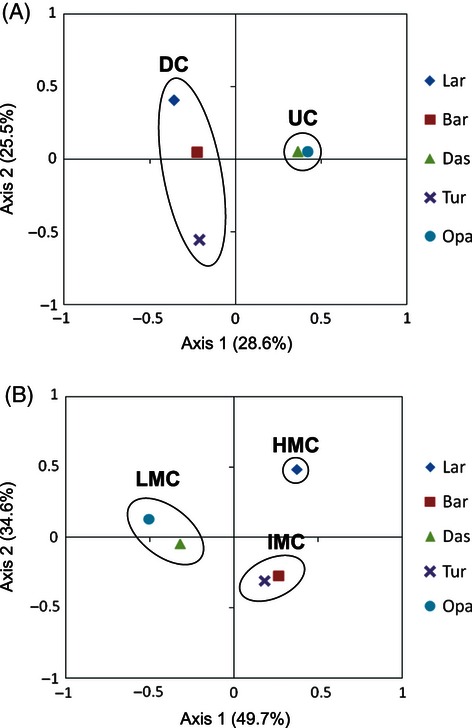
Communities clustered at the membership level and the composition level using PCoA of the unweighted UniFrac distance matrix. Communities clustered at (A) the membership level (taxonomic diversity) and (B) the composition level (taxonomic structure) using PCoA of the unweighted UniFrac distance matrix. In (A), two groups are showed, the disturbed communities (DC) and the undisturbed communities (UC). In (B), three groups are showed following the polymetallic contamination magnitude, namely the low metallic contaminated (LMC) communities, the intermediate metallic contaminated (IMC) communities and the high metallic contaminated (HMC) communities. Contaminated reference site: Turcotte Lake (Tur); clean reference site: Opasatica Lake (Opa); tested lake system: Arnoux Lake (Lar), Arnoux Bay (Bar), and Dasserat Lake (Das).

Principal coordinate analyses (PCoA) of the unweighted (diversity) and weighted (structure) UniFrac distance matrix are showed in Fig. 4. Considering the diversity level, PCoA of the unweighted Unifrac distance matrix provided compelling evidences about the clustering of the undisturbed communities (UC), namely Das and Opa, and the clustering of the disturbed communities (DC) Lar-Bar-Tur. Through PCoA, the weighted Unifrac distance matrix clearly demonstrated that the communities' structure clustered in three groups consistent with the magnitude of the metallic contamination. The first node clustered the LMC communities Opa and Das, while the second cluster closely grouped the IMC communities Tur and Bar. Considering its unique taxonomical structure, Lar was the sole representative of the third cluster identified as a HMC community.

### Taxonomical survey at the diversity level

**Figure 5:**
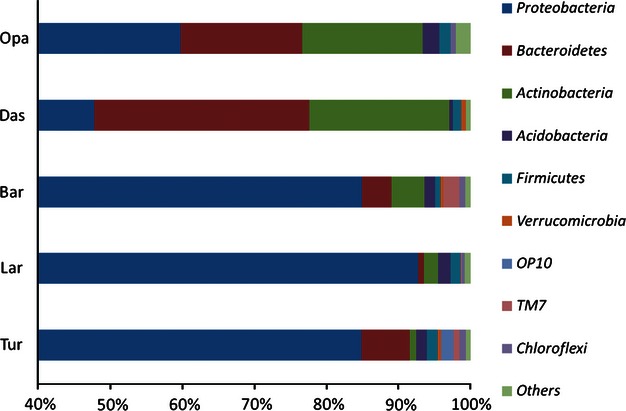
Variation of the principal phyla relative OTUs abundance between communities. Clean reference site: Opasatica Lake (Opa); contaminated reference site: Turcotte Lake (Tur); tested lake system: Arnoux Lake (Lar), Arnoux Bay (Bar), and Dasserat Lake (Das). Phyla regrouped under the term *Others* are the following: Opa = *Chlorobi, Gemmatimonadetes, Lentisphaerae, Spirochaetes,* and *Synergistetes*; Tur = *Caldiserica* and *Chlorobi*; Das = *Deferribacteres, Gemmatimonadetes,* and *Lentisphaerae*; Bar = *Aquificae* and *Planctomycetes*; Lar = *Chlorobi*, *Nitrospira,* and *Spirochaetes*.

Based on each phylum's relative OTUs abundance, the taxonomical survey at the diversity level (see Fig. 5) revealed that the *Proteobacteria* were prevalent among the disturbed communities. Deeper analysis at the class level among the *Proteobacteria* exerts the high relative proportion of OTUs belonging to the *Alphaproteobacteria* (Lar = 64.30% > Tur = 56.93% > Bar = 33.58% > Opa = 30.66% > Das = 15.43%), followed by the *Betaproteobacteria* (Bar = 34.34% > Das = 25.76% > Opa =16.25% >Lar = 16.17% > Tur = 13.20%). The second most dominant phylum based on the relative OTUs abundance was represented by the *Bacteroidetes*, which are found primarily in the undisturbed sites and mainly encompassed the classes *Flavobacteria* (Das = 18.35% > Opa = 8.47% > Tur = 0.65% > Bar = 0.38% > Lar = undetected) and *Sphingobacteria* (Das = 9.48% > Opa = 7.78% > Tur = 5.41% > Bar = 3.02% > Lar = 0.59%). The third phylum that showed high degree of OTU diversity was *Actinobacteria* which, as for the *Bacteroidetes*, exhibited higher OTU diversity within the noncontaminated sites. Among the *Actinobacteria*, most representatives were regrouped in the class of the *Actinomycetales* (Das = 16.04% > Opa = 10.30% > Bar = 3.40% > Lar = 1.18% > Tur = 0.43%) followed by the class *Acidimicrobiales* (Das = 1.58% > Lar = 0.79% > Bar = 0.75% > Tur = 0.43% > Opa = 0.23%). Several other phyla were identified within the studied system such as the *Acidobacteria*, *Firmicutes*, *TM7*, *Chloroflexi*, *OP10* and *Verrucomicrobia*, but they showed very weak relative OTU abundances (less than 2.29% in at least one site).

The relative OTUs abundance survey permitted establishing the ten most dominant genera at the diversity level within each community (see Table 4). The genus *Polynucleobacter* (class *Betaproteobacteria*) showed greatest relative abundance (Bar =28.30% > Lar = 11.83% > Das = 8.87% > Tur = 6.06% > Opa = 5.26%). The second best represented genus was *Kozakia* from the class *Alphaproteobacteria* (Lar = 19.33% > Bar = 0.38% > Das = 0.24% > Tur-Opa = undetected), followed by the *Acidisphaera* (Lar = 13.21% > Bar = 1.89% > Tur = 0.87% > Das = 0.12% > Opa = undetected), the genus *Flavobacterium* from the *Bacteroidetes* phylum (Opa = 8.47% > Das = 6.93% > Bar = 0.38% > Tur-Lar = undetected), and the genus *Citreimonas* from the class *Alphaproteobacteria* (Tur = 14.07% > Opa = 0.23% > Das-Bar-Lar = undetected).

**Table 4:** Survey of the ten dominant genera found within each community considering the taxonomical diversity (based on the relative OTUs abundance)

Site	Genera	Relative OTUs abundance (%)	Site	Genera	Relative OTUs abundance (%)
Lar	*Kozakia*	19.33	Tur	*Citreimonas*	14.07
	*Acidisphaera*	13.21		*Fodinicurvata*	7.36
	*Polynucleobacter*	11.83		*Polynucleobacter*	6.06
	*Acidocella*	10.06		*Tanticharoenia*	2.81
	*Rhodovarius*	3.35		*Sediminibacterium*	1.95
	*Tanticharoenia*	2.56		*OP10 Unclassified*	1.73
	*Rhodopila*	2.17		*Ponticoccus*	1.52
	*Legionella*	1.58		*Wenxinia*	1.52
	*Acidobacteria_GP1*	0.79		*Agromonas*	1.30
	*Acidiphilium*	0.79		*Rhodopila*	1.08
Bar	*Polynucleobacter*	28.30	Opa	*Ilumatobacter*	5.49
	*Novosphingobium*	4.15		*Polynucleobacter*	5.26
	*Cohaesibacter*	3.40		*Oryzihumus*	4.12
	*TM7 Unclassified*	2.64		*Flavobacterium*	3.20
	*Acidocella*	1.89		*Legionella*	3.20
	*Acidisphaera*	1.89		*Rhodoferax*	2.52
	*Beijerinckia*	1.51		*Novosphingobium*	2.29
	*Arsenicicoccus*	1.13		*Pelagibacter*	1.83
	*Rhodoferax*	1.13		*Zunongwangia*	1.37
	*Nubsella*	1.13		*Laribacter*	1.37
Das	*Polynucleobacter*	8.87			
	*Flavobacterium*	6.93			
	*Zunongwangia*	4.62			
	*Arcicella*	3.40			
	*Lapillicoccus*	3.04			
	*Persicobacter*	2.79			
	*Fodinibacter*	2.43			
	*Rhodoferax*	1.70			
	*Fulvivirga*	1.70			
	*Intrasporangium*	1.58			

Lar, Arnoux Lake; Bar, Arnoux Bay; Das, Dasserat Lake; Tur, Turcotte Lake; Opa, Opasatica Lake.

### Taxonomical survey at the structure level

**Figure 6:**
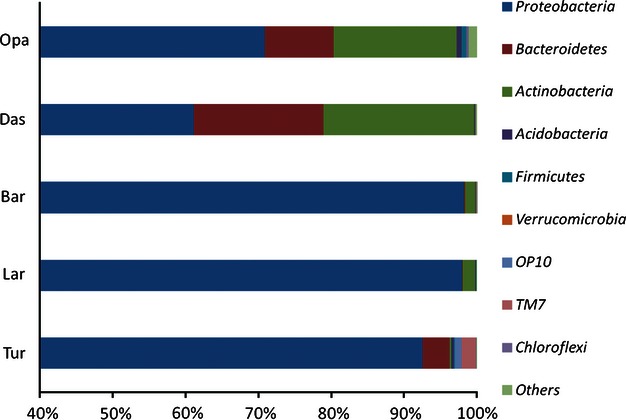
Variation of the principal phyla relative sequences abundance between communities. Clean reference site: Opasatica Lake (Opa); contaminated reference site: Turcotte Lake (Tur); tested lake system: Arnoux Lake (Lar), Arnoux Bay (Bar), and Dasserat Lake (Das). Phyla regrouped under the term *Others* are the following: Opa = *Chlorobi, Gemmatimonadetes, Lentisphaerae, Spirochaetes,* and *Synergistetes*; Tur = *Caldiserica* and *Chlorobi*; Das = *Deferribacteres, Gemmatimonadetes,* and *Lentisphaerae*; Bar = *Aquificae* and *Planctomycetes*; Lar = *Chlorobi*, *Nitrospira,* and *Spirochaetes*.

Based on each phylum's relative sequences abundance, the taxonomical survey at the structure level (see Fig. 6) revealed that the *Proteobacteria* are dominant among disturbed communities (Lar = 97.94% > Bar = 98.21% > Tur =92.53% > Opa = 70.86% > Das = 61.16%). Deeper analysis within the *Proteobacteria* phylum revealed the high relative proportion of sequences belonging to the *Betaproteobacteria* (Bar = 94.82% > Tur = 36.13% > Das = 35.06% > Opa = 27.45% > Lar = 19.79%), followed by the *Alphaproteobacteria* (Lar = 77.51% > Tur = 54.68% > Opa = 38.99% > Das = 24.02% > Bar = 2.94%). The second most dominant phylum based on the relative sequences abundance was represented by the *Actinobacteria,* which contained higher OTU diversity within the noncontaminated sites (Das = 20.66% > Opa = 16.90% > Lar = 1.65% > Bar = 1.37% > Tur = 0.20%). Among the *Actinobacteria*, most representatives were regrouped in the class of the *Actinomycetales* (Das = 19.60% > Opa = 2.06% >Lar = 1.61% > Bar = 1.33% > Tur = 0.17%).

The third phylum exposing the broader structural complexity was the *Bacteroidetes*, which were found mostly in the undisturbed sites (Das = 17.78% > Opa = 9.44% > Tur = 3.74% > Bar = 0.16% > Lar = 0.15%) and mainly regrouped two classes: *Flavobacteria* (Das = 12.22% > Opa = 4.08% > Tur = 0.05% > Bar = 0.02% > Lar = undetected) and *Sphingobacteria* (Das = 4.85% > Opa = 2.97% > Tur = 3.64% > Lar = 0.14% > Bar = 0.13%).

The survey of the relative sequences abundance allowed establishing the ten most structurally dominant genera within each community (see Table 5). The genus *Polynucleobacter* (class *Betaproteobacteria*) prevailed within all the communities (Bar = 94.55% > Tur = 34.34% > Das = 28.74% > Lar = 19.55% > Opa = 16.61%). The second genus with the highest representatives was *Kozakia* from the class *Alphaproteobacteria* (Lar = 47.66% > Das = 3.53% > Bar = 1.02% > Tur-Opa = undetected), followed by the genus *Pelagibacter* (Opa = 22.09% > Das = 18.57% > Bar = 5.09% > Das-Tur = undetected) and the genus *Fodinicurvata* (Tur = 33.37% > Bar = 1.02% > Das-Bar-Lar = undetected).

**Table 5:** Survey of the ten dominant genera found within each community considering the taxonomical structure (based on the relative sequences abundance)

Site	Genera	Relative sequences abundance (%)	Site	Genera	Relative sequences abundance (%)
Lar	*Kozakia*	47.66	Tur	*Polynucleobacter*	34.34
	*Polynucleobacter*	19.55		*Fodinicurvata*	33.37
	*Acidocella*	15.63		*Rhodopila*	9.06
	*Acidisphaera*	11.11		*Citreimonas*	2.27
	*Rhodovarius*	1.57		*TM7 unclassified*	2.02
	*Oryzihumus*	1.51		*Segetibacter*	1.91
	*Rhodopila*	0.42		*Tanticharoenia*	1.51
	*Nubsella*	0.13		*Sediminibacterium*	1.46
	*Tanticharoenia*	0.12		*Zhangella*	1.37
	*Legionella*	0.10		*Pleomorphomonas*	1.12
Bar	*Polynucleobacter*	94.55	Opa	*Pelagibacter*	22.09
	*Arsenicicoccus*	1.18		*Polynucleobacter*	16.61
	*Acidocella*	0.63		*Pseudorhodoferax*	5.89
	*Novosphingobium*	0.61		*Oryzihumus*	5.36
	*Beijerinckia*	0.22		*Ilumatobacter*	4.60
	*Agromonas*	0.16		*Dermabacter*	2.39
	*Cohaesibacter*	0.15		*Alkaliflexus*	2.33
	*Acidisphaera*	0.14		*Beijerinckia*	2.27
	*Afipia*	0.13		*Caulobacter*	1.69
	*TM7 Unclassified*	0.09		*Zunongwangia*	1.46
Das	*Polynucleobacter*	28.74			
	*Flavobacterium*	21.88			
	*Pelagibacter*	18.57			
	*Fodinibacter*	11.72			
	*Microterricola*	3.21			
	*Rhodoferax*	2.98			
	*Arcicella*	2.75			
	*Beijerinckia*	1.48			
	*Persicobacter*	1.46			
	*Janibacter*	1.20			

Lar, Arnoux Lake; Bar, Arnoux Bay; Das, Dasserat Lake; Tur, Turcotte Lake; Opa, Opasatica Lake.

### Physicochemical correlation analyses at the diversity level

All the correlation results gained from anova analyses between the diversity data collected and the physicochemical parameters measured at each site are summarized in Tables 3 and 6. Among the diversity indices recovered using Mothur, only Shannon indices revealed a significant correlation with the dissolved organic carbon (DOC) measures (*R*^2^ = 0.90; *P* = 0.01379).

**Table 6:** Survey of the significant correlations found considering the OTUs of each community and defining the communities' diversity

	Al	Cd	Cu	Fe	Pb	Zn	Mn	Na	S	Ca	Mg	DOC	pH	Temp.
*H'*	NS	NS	NS	NS	NS	NS	NS	NS	NS	NS	NS	*(0.90)	NS	NS
P1	NS	†(0.95)	NS	NS	NS	NS	NS	NS	NS	NS	NS	NS	*(0.87)	NS
P1.1	NS	*(0.88)	NS	NS	NS	NS	NS	NS	NS	NS	NS	NS	NS	NS
P2	NS	*(0.90)	NS	NS	NS	NS	NS	NS	NS	NS	NS	NS	*(0.88)	NS
P2.1	NS	*(0.85)	NS	NS	NS	NS	NS	NS	NS	NS	NS	NS	NS	NS
P3	NS	NS	NS	NS	NS	NS	NS	NS	NS	NS	NS	*(0.78)	NS	NS
P4	NS	NS	NS	NS	NS	NS	NS	NS	NS	*(0.90)	NS	NS	NS	NS
G1	†(0.93)	NS	*(0.91)	‡(1.00)	†(0.98)	*(0.89)	*(0.81)	NS	*(0.81)	NS	NS	NS	NS	†(0.93)
G2	*(0.85)	NS	*(0.82)	‡(0.98)	‡(1.00)	*(0.79)	NS	NS	NS	NS	NS	NS	NS	*(0.86)
G3	†(0.95)	NS	‡(1.00)	*(0.87)	*(0.80)	‡(0.99)	†(0.93)	NS	*(0.91)	NS	NS	NS	*(0.77)	†(0.98)
G4	†(0.96)	NS	†(0.92)	‡(1.00)	†(0.96)	*(0.91)	*(0.85)	NS	*(0.87)	NS	NS	NS	NS	†(0.94)
G5	NS	NS	NS	NS	NS	NS	NS	NS	NS	*(0.90)	NS	NS	NS	NS
G6	NS	NS	NS	NS	NS	NS	NS	NS	NS	*(0.90)	NS	NS	NS	NS
G7	NS	NS	NS	NS	NS	NS	NS	*(0.90)	NS	NS	NS	NS	NS	NS
G8	†(0.86)	NS	*(0.83)	‡(0.99)	‡(1.00)	*(0.80)	NS	NS	NS	NS	NS	NS	NS	*(0.88)
G9	NS	*(0.82)	NS	NS	NS	NS	NS	NS	NS	NS	NS	*(0.81)	*(0.90)	NS
G10	NS	NS	NS	NS	NS	NS	NS	NS	NS	*(0.89)	*(0.85)	NS	NS	NS
G11	NS	NS	NS	NS	NS	NS	NS	*(0.89)	NS	NS	NS	NS	NS	NS
G12	NS	*(0.83)	NS	NS	NS	NS	NS	*(0.79)	NS	NS	NS	NS	*(0.85)	NS
G13	NS	NS	NS	NS	NS	NS	NS	NS	NS	*(0.90)	NS	NS	NS	NS
G14	NS	NS	* (0.86)	NS	NS	*(0.83)	NS	NS	NS	NS	NS	NS	*(0.88)	*(0.85)
G15	†(0.95)	NS	†(0.94)	‡(0.99)	†(0.96)	†(0.92)	*(0.85)	NS	*(0.88)	NS	NS	NS	NS	†(0.96)
G16	NS	*(0.82)	NS	NS	NS	NS	NS	NS	NS	NS	NS	NS	NS	NS
G17	NS	NS	NS	NS	NS	NS	NS	NS	NS	NS	NS	*(0.78)	NS	NS
G18	NS	NS	NS	NS	NS	NS	NS	NS	NS	*(0.78)	NS	NS	NS	NS

* *P*-value between 0.01 and 0.05.

† *P*-value between 0.001 and 0.01.

‡ *P*-value between 0 and 0.001.

NS = *P*-value ≥0.05; (*R*^2^).

Data highlighted in grey are negative correlations. The column for potassium was removed since no significant correlations were found.

Al, Aluminum; Ca, Calcium; Cd, Cadmium; Cu, Copper; Fe, Iron; Mg, Magnesium; Mn, Manganese; Na, Sodium; Pb, Lead; S, Sulfur; Zn, Zinc; DOC, Dissolved Organic Carbon; Temp., Temperature; *H'*, Shannon Index.

Phyla: P1 = *Actinobacteria*, P1.1 = *Actinomycetales*, P2 = *Proteobacteria*, P2.1 = *Alphaproteobacteria*, P3 = *TM7*, P4 = *OP10*; Genera: G1 = *Acidisphaera*, G2 = *Acidiphilium*, G3 = *Acidobacteria_GP1*, G4 = *Acidocella*, G5 = *Citreimonas*, G6 = *Fodinicurvata*, G7 = *Ilumatobacter*, G8 = *Kozakia*, G9 = *Laribacter*, G10 = *OP10_Unclassified*, G11 = *Orizihumus*, G12 = *Pelagibacter*, G13 = *Ponticoccus*, G14 = Rhodoferax, G15 = *Rhodovarius*, G16 = *Tanticharoenia*, G17 = *TM7_Unclassified*, G18 = *Wenxinia*.

At the phylum level, statistical analyses also revealed that the relative abundance of OTUs belonging to the *Actinobacteria* was negatively influenced by the cadmium gradient (negative correlation, *R*^2^ = 0.95; *P* = 0.005365) while favored by near neutral pH measures (*R*^2^ = 0.87, *P* = 0.02067). Interestingly, the most diverse order within the class *Actinobacteria*, namely the *Actinomycetales*, mainly explained such a negative correlation with cadmium (negative correlation, *R*^2^ = 0.88; *P* = 0.01725). On the reciprocal, the diversity of the phylum *Proteobacteria* was significantly correlated with cadmium concentrations (*R* = 0.90; *P* = 0.01417) and negatively influenced by alkaline pH environments (negative correlation, *R*^2^ = 0.88, *P* = 0.01753). Deeper analysis among the *Proteobacteria* revealed that the *Alphaproteobacteria* was the sole class showing significant correlations with cadmium measures (*R*^2^ = 0.85; *P* = 0.02718). Moreover, the phylum OP10 exhibited a significant negative correlation with calcium concentrations (negative correlation, *R*^2^ = 0.90; *P* = 0.01395) and the phylum TM7 was negatively correlated with DOC values (negative correlation, *R*^2^ = 0.78; *P* = 0.04813).

At the genus level, trends indicated that the interconnected and disturbed communities of Lar and Bar shared two genera with similar co-tolerances to various metalloids: *Acidisphaera* and *Acidocella*. Furthermore, the community of Lar showed additional significant correlations as revealed by the genera *Acidiphilium*, *Acidobacteria_GP1 Unclassified*, *Kozakia* and the genus *Rhodovarius*. Interestingly, the Lar bacterial consortium hosted the genus *Tanticharoenia* which was also found in Tur, the reference community for contamination.

### Physicochemical correlation analyses at the structure level

The results recovered from anova analyses at the structure level by assessing the correlations between the relative sequences abundance and the physicochemical parameters are summarized in Table 7. At the structure level, the correlations found considering the dominant phyla were comparable to the ones found at the diversity level, with the exception that additional correlations were found with DOC. Therefore, the relative abundance of sequences belonging to the *Actinobacteria* was negatively influenced by the cadmium gradient, while favored by higher dissolved organic carbon content and neutral pH (Cd: negative correlation, *R*^2^ = 0.89; *P* = 0.01605, DOC: *R*^2^ = 0.80; *P* = 0.04009, pH: *R*^2^ = 0.85; *P* = 0.02522). Within the class *Actinobacteria*, the *Actinomycetales* still mainly explained such a negative correlation with cadmium (negative correlation, *R*^2^ = 0.82; *P* = 0.03407). As found at the diversity level, the structure of the phylum *Proteobacteria* significantly correlated with cadmium concentrations, and negatively correlated with alkaline pH environments and high DOC values (Cd: *R*^2^ = 0.82; *P* = 0.03325, DOC: negative correlation, *R*^2^ = 0.80; *P* = 0.04116, pH: negative correlation, *R*^2^ = 0.86; *P* = 0.02199). Interestingly, the class *Alphaproteobacteria* did not support the correlation found between the structure of the phylum *Proteobacteria* and the cadmium gradient as observed at the diversity level.

**Table 7:** Survey of the significant correlations found considering the sequences of each community and defining the communities' structure

	Al	Cd	Cu	Fe	Pb	Zn	Na	Ca	K	Mg	DOC	PH =	Temp.
P1	NS	*(0.89)	NS	NS	NS	NS	NS	NS	NS	NS	*(0.80)	*(0.85)	NS
P1.1	NS	*(0.82)	NS	NS	NS	NS	NS	NS	NS	NS	NS	NS	NS
P2	NS	*(0.82)	NS	NS	NS	NS	NS	NS	NS	NS	*(0.80)	*(0.86)	NS
P3	NS	NS	NS	NS	NS	NS	NS	*(0.90)	NS	NS	NS	NS	NS
P4	NS	NS	NS	NS	NS	NS	NS	*(0.90)	NS	NS	NS	NS	NS
P5	NS	NS	NS	NS	NS	NS	*(0.88)	NS	NS	NS	NS	NS	NS
P6	NS	NS	NS	NS	NS	NS	*(0.84)	NS	NS	NS	NS	NS	NS
G1	*(0.86)	NS	*(0.83)	‡(0.99)	‡(1.00)	*(0.80)	NS	NS	NS	NS	NS	NS	*(0.87)
G2	*(0.88)	NS	*(0.85)	‡(1.00)	‡ (1.00)	*(0.82)	NS	NS	NS	NS	NS	NS	*(0.89)
G3	NS	NS	NS	NS	NS	NS	*(0.91)	NS	NS	*(0.81)	NS	NS	NS
G4	NS	NS	NS	NS	NS	NS	†(0.94)	NS	NS	NS	NS	NS	NS
G5	NS	*(0.87)	NS	NS	NS	NS	NS	NS	NS	NS	NS	†(0.92)	NS
G6	NS	NS	NS	NS	NS	NS	†(0.94)	NS	NS	NS	NS	NS	NS
G7	NS	NS	NS	NS	NS	NS	NS	*(0.90)	NS	†(0.77)	NS	NS	NS
G8	NS	NS	NS	NS	NS	NS	†(0.94)	NS	NS	NS	NS	NS	NS
G9	NS	NS	NS	NS	NS	NS	NS	*(0.90)	NS	NS	NS	NS	NS
G10	NS	NS	NS	NS	NS	NS	†(0.93)	NS	NS	NS	NS	NS	NS
G11	*(0.85)	NS	*(0.82)	‡(0.98)	‡ (1.00)	†(0.79)	NS	NS	NS	NS	NS	NS	*(0.86)
G12	NS	NS	NS	NS	NS	NS	*(0.90)	NS	NS	NS	NS	NS	NS
G13	NS	NS	NS	NS	NS	NS	*(0.91)	NS	*(0.77)	NS	NS	NS	NS
G14	NS	NS	NS	NS	NS	NS	†(0.92)	NS	NS	NS	NS	NS	NS
G15	NS	*(0.89)	NS	NS	NS	NS	NS	NS	NS	NS	*(0.84)	†(0.93)	NS
G16	NS	NS	NS	NS	NS	NS	NS	*(0.90)	NS	*(0.78)	NS	NS	NS
G17	NS	NS	NS	NS	NS	NS	†(0.94)	NS	NS	NS	NS	NS	NS
G18	NS	NS	NS	NS	NS	NS	NS	*(0.87)	NS	NS	NS	NS	NS
G19	*(0.86)	NS	*(0.83)	‡(0.99)	‡(1.00)	*(0.80)	NS	NS	NS	NS	NS	NS	*(0.88)
G20	NS	NS	NS	NS	NS	NS	NS	†(0.93)	NS	*(0.82)	NS	NS	NS
G21	NS	NS	NS	NS	NS	NS	NS	*(0.91)	NS	*(0.78)	NS	NS	NS
G22	NS	NS	NS	NS	NS	NS	NS	*(0.85)	NS	NS	NS	NS	NS
G23	NS	NS	NS	NS	NS	NS	NS	*(0.90)	NS	NS	NS	NS	NS
G24	NS	NS	NS	NS	NS	NS	NS	*(0.90)	NS	*(0.77)	NS	NS	NS
G25	NS	*(0.86)	NS	NS	NS	NS	NS	NS	NS	NS	*(0.83)	†(0.93)	NS

* *P*-value between 0.01 and 0.05.

† *P*-value between 0.001 and 0.01.

‡ *P*-value between 0 and 0.001.

NS = *P*-value ≥0.05; (*R*^2^).

Data highlighted in grey are negative correlations. The column for potassium was removed since no significant correlations were found.

Al, Aluminum; Ca, Calcium; Cd, Cadmium; Cu, Copper; Fe, Iron; K, Potassium; Mg, Magnesium; Mn, Manganese; Na, Sodium; Pb, Lead; Zn, Zinc; DOC, Dissolved Organic Carbon; Temp., Temperature.

Phyla: P1 = *Actinobacteria*, P1.1 = *Actinomycetales*, P2 = *Proteobacteria*, P3 = *TM7*, P4 = *OP10*, P5 = *Firmicutes*, P6 = *Chloroflexi*; Genera: G1 = *Acidisphaera*, G2 = *Acidocella*, G3 = *Agromonas*, G4 = *Alkaliflexus*, G5 = *Beijerinckia*, G6 = *Caulobacter*, G7 = *Citreimonas*, G8 = *Dermabacter*, G9 = *Fodinicurvata*, G10 = *Ilumatobacter*, G11 = *Kozakia*, G12 = *Legionella*, G13 = *Nubsella*, G14 = *Oryzihumus*, G15 = *Pelagibacter*, G16 = *Pleomorphomonas*, G17 = *Pseudorhodoferax*, G18 = *Rhodopila,* G19 = *Rhodovarius,* G20 = *Sediminibacterium,* G21 = *Tanticharoenia,* G22 = *Segetibacter,* G23 = *Rhodopila,* G24 = *TM7_Unclassified,* G25 = *Zunongwangia*.

At the genus level, Lar and Bar still shared the two same genera correlated with multiple metalloids, namely the *Acidisphaera* and the *Acidocella*. Moreover, Lar community still showed significant correlations considering the genera *Kozakia* and *Rhodovarius*. However, no more correlation with cadmium was found for the disturbed communities of Lar and Tur. In contrast, the undisturbed community of Opa still showed a negative correlation with cadmium considering the genus *Pelagibacter*, a genus also shared with the undisturbed community of Das. Moreover, an additional negative correlation between cadmium and the relative sequences abundance of the genus *Beijerinckia* was found for Opa, and shared with Das and Bar communities. The genus *Zunongwangia*, newly retrieved from the community of Opa, also exposed such a negative influence induced by the cadmium content.

Interphylum correlation analyses using the relative OTUs abundances revealed that the species diversity between the phyla *Proteobacteria* and *Bacteroidetes* was negatively correlated (negative correlation, *R*^2^ = 0.96; *P* = 0.003002), as for the *Proteobacteria* and the *Actinobacteria* (negative correlation, *R*^2^ = 0.95; *P* = 0.00478), while a significant positive correlation was detected between the OTUs diversity of the *Bacteroidetes* and the *Actinobacteria* (*R*^2^ = 0.86; *P* = 0.02321). Considering the communities' structure as assessed by the relative sequence abundance for each OTU, similar interphylum correlations were observed. In this respect, the structure of the phyla *Proteobacteria* and *Bacteroidetes* were negatively correlated (negative correlation, *R*^2^ = 0.95; *P* = 0.004256), as for the *Proteobacteria* and the *Actinobacteria* (negative correlation, *R*^2^ = 0.96; *P* = 0.003340), while a significant positive correlation was detected between the structure of the *Bacteroidetes* and the *Actinobacteria* (*R*^2^ = 0.87; *P* = 0.02152).

### Bacterial network analysis

**Figure 7:**
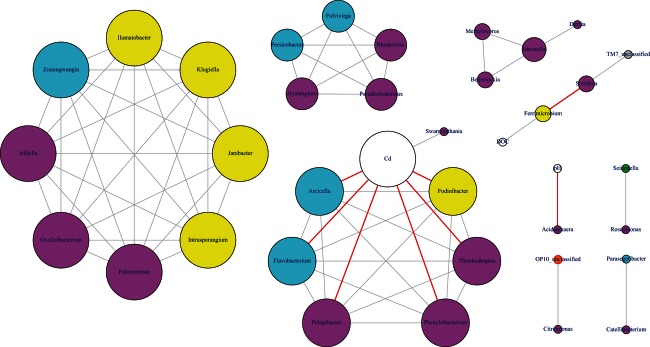
Bacterial networks of the 50 dominant species and the physicochemical parameters of interest (Cd = cadmium; DOC = Dissolved organic carbon; pH = pH measure). The nodes represents the OTUs with 97% identity at a *P*-value of <0.01 and each node color indicates to which phylum the OTU belongs (yellow = *Actinobacteria*, blue = *Bacteroidetes*, purple = *Proteobacteria*, green = *Firmicutes*, orange = *OP10,* and grey = *TM7*). Each strongly significant correlation is an edge represented by a grey line. Edges indicative of a negative significant correlation are represented by a red line.

Among the 50 dominant species, only 31 exhibited strong and significant correlations. Based on these significant correlations, three abiotic parameter-associated networks and six bacteria-exclusive networks were built (see Fig. 7). The first abiotic parameter-associated network identifies cadmium, which was found to be positively correlated with the OTU *Swaminathani*a, while negatively correlated with the abundance of the species *Fodinibacter*, *Thiorhodospira*, *Phenylobacterium* and *Pelagibacter*, *Flavobacterium,* and *Arcicella*. The second parameter-associated network identifies the DOC parameter positively correlated with the relative abundance of *Ferrimicrobium*. *Ferrimicrobium* was found to be negatively correlated with the *Simiduia* abundance, which in turn was positively correlated with the *TM7_unclassified* OTU. The third abiotic parameter-associated network put in evidence the negative correlation between the pH parameter and the abundance of *Acidisphaera*. Besides these particular abiotic parameter-associated networks, networks based exclusively on correlations found between bacterial species were also identified. The first bacteria-exclusive network regrouped OTUs of the *Actinobacteria* (*Ilumatobacter*, *Klugiella*, *Janibacter,* and *Intrasporangium*), few members of the Proteobacteria (*Polaromonas*, *Oxalicibacterium,* and *Afifella*) and a sole representative of the *Bacteroidetes* (*Zunongwagia*). The second bacteria-exclusive network regrouped species from the *Actinobacteria* (*Rhodoferax*, *Pseudorhodoferax,* and *Hyalangium*) and members of the *Bacteroidetes* (*Fulvivirga* and *Persicobacter*). The third bacteria-exclusive network involved only *Actinobacteria* member interactions, namely with OTUs *Methylovorus*, *Beijerinckia*, *Ideonella,* and *Derxia*. The three last bacteria-exclusive networks consisted in single correlations between two species: *Seinonella* - *Roseomonas*, *OP10_unclassified* - *Roseomonas*, and *Parasegetibacter* - *Catellibacterium*.

## Discussion

Through the identification of taxonomic interactions impacted by similar selective pressures, our study aimed to shed light on the importance of analyzing communities from a phylogenetic perspective. However, we also demonstrated that the inclusion of a quantitative approach provides even deeper insight into the evolutionary forces that shape the dynamics of key taxonomic interaction networks in bacterial communities. Our first objective was to assess whether natural selection drove parallel adaptation at the community taxonomical diversity and structure levels on two geographically isolated bacterial communities under similar selective pressure. The second objective was to test whether a polymetallic gradient exposure in three interconnected lacustrine bacterial communities triggered significant taxonomical membership and abundance shifts. Finally, the third objective was to identify the related environmental factor driving community changes in the studied system.

Despite recent progress in characterizing the diversity of freshwater microorganisms, drivers of changes in community composition have not yet been well characterized. Anthropogenic environmental disturbance may have a great impact on the functional diversity of bacterial processes. Given such disturbances are increasingly common, there is a need for accurate predictive models regarding their impact on bacterial community ecology and taxomonic diversity (Konopka 2009). More specifically, as taxonomic diversity is suspected to influence the metabolic pathway interacting networks, a first step is to identify key taxonomic interaction networks that are lost in the face of strong environmental perturbation. Using high-throughput sequencing of 16S ribosomal subunit gene libraries, this study successfully identified environmental parameters with the power to drive key taxonomic interaction networks in bacterial communities, both at the community membership (diversity) and composition (structure) levels. In doing so, we characterized the impact of a polymetallic contamination gradient over a system of both independent and interconnected lacustrine bacterial communities.

Among the collected data, geochemical analysis of lake water samples indicated that the heavy metal content in Opa was similar to the one found in Das, while the heavy metal concentrations in Tur were similar to those measured in Bar. Consistent with the geochemical data, PCoA, Venn and phylogenetic analyses at the microbial diversity level supported the clustering of undisturbed communities on one side and disturbed communities on the other. In this respect, the clustering of the geographically isolated community of Tur with the interconnected communities of Bar and Lar revealed that parallel adaptation can occur at the taxonomic level between independent communities if impacted by a similar selective pressure. Such a parallel change of interacting taxonomical networks suggests that community adaptation is the net effect of individual members' successful and unsuccessful acclimation/adaptation to environmental perturbation, as proposed by DeAngelis et al. (2010). Therefore, parallel changes of interacting taxonomical networks would suggest in turn that HGT of loci corresponding to heavy metal resistance were, at the most, limited to closely related species, as argued by Andam and Gogarten (2011). Thus, our results suggest that two independent bacterial communities living in similar environmental conditions exhibit the same overall taxonomical membership (i.e. members in both communities belonging to the same high taxonomic ranks).

At the structure level (i.e. OTU relative abundance), PCoA, Venn and phylogenetic analyses supported the clustering of the communities into three groups according to the cadmium gradient, thus exerting the fact that cadmium was the most lethal heavy metal measured in this study. As such, in relation to relative taxon abundance, weighted structural analyses grouped communities Tur and Bar into an intermediate metallic contaminated communities cluster and further demonstrated that higher cadmium concentrations thoroughly disturbed Lar community structure (Fig. 4B). This result was striking because Lar and Bar lakes are connected. Overall, these results first bring to light evidence that community differentiation at the taxonomical diversity level reflects the presence/absence of a perturbation, while the community structure level accurately reflects the magnitude of the perturbation.

Our study has therefore enlightened how parallel community adaptation resulting from a similar environmental perturbation may have occurred within the *original core microbiome* due to selection of a *heavy metal resistant core* OTUs combined to the loss of most sensitive bacterial species. Consequently, all the OTUs shared across the five lakes may be considered as part of the *original core microbiome*. Then, the OTUs shared between Opa and Das may be identified as the *healthy core microbiome* mirroring the absence of contamination while the persistent species shared between the polluted sites make up the *heavy metal resistant core microbiome* selected from the *original core microbiome*. A comparable bacterial community adaptation pattern was observed for copper contamination, where membership shifts translated by an increase of *Proteobacteria* (Turpeinen et al. 2004). In this study, a similar trend was observed: *Proteobacteria* were widely prevalent among the disturbed communities, accounting for more than 84% of the OTUs and their relative abundance reached more than 92% of the sequences, while their proportion dropped to less than 60% of the OTUs and their abundance was less than 71% of the sequences in the undisturbed communities. As revealed by the correlation analyses, the diversity among the *Proteobacteria* was mainly favored by higher cadmium level and acidic pH values. The *Proteobacteria* dominance was also influenced by these two previous factors, and by the dissolved organic content. Such finding could be explained by either the presence of heavy metals resistant species among the *Proteobacteria* and/or the fact that this particular phylum encompasses more generalist species, thus conferring competitive traits to the whole phylum in case of a perturbation (Barberán et al. 2012). Furthermore, the *Alphaproteobacteria* OTUs diversity was found to underlie such a correlation between *Proteobacteria* and cadmium. Interestingly, among the *Alphaproteobacteria*, several genera showed positive correlations with Fe, the highest metalloid measured in the disturbed sites, but also the most biologically important metal cation owing to its importance in the electron transport chain (Nies 1999). Furthermore, numerous *Alphaproteobacteria* genera exhibited positive correlations with Al, Cu, Pb, Zn, and Mn. This observation illustrates Alphaproteobacterial resiliance to polymetallic contamination and may account for the high Chao richness and the number of OTUs observed (*S*_obs_) in Lar. Alphaproteobacterial genera encompassed *Acidiphilium*, *Acidimicrobium*, *Acidisphaera*, *Acidocella*, *Kozakia,* and *Rhodovarius*, which were found to be favored at both the diversity and structure levels in Lar. Interestingly, the *Alphaproteobacteria* are known to be highly successful aquatic lineages. Competitive at low nutrient levels and resistant to grazing by organisms of higher trophic levels (Newton et al. 2011), they also bear genomes enriched with genes belonging to xenobiotic degradation pathways (Debroas et al. 2009). Moreover, heterotrophic acidophiles belonging to the *Alphaproteobacteria* class such as the genera *Acidiphilium, Acidocella, and Acidomicrobium* appeared to be widely distributed in metal-rich acidic environments as they catalyze processes implied in the iron cycling (Harrison 1984; Johnson and McGinness 1991; Küsel et al. 1999, 2002; Hallberg and Johnson 2001, 2003). Our results therefore confirm the overall opportunistic ability of the *Proteobacteria*, particularly the class of *Alphaproteobacteria*, commonly identified in lake epilimnion.

The phylum *Actinobacteria* is also highly abundant in freshwater environments (Newton et al. 2007, 2011). In this study, the phylum *Actinobacteria* was severely impaired both at the diversity and structure levels, by exposure to cadmium, acidic pH, and low DOC content, especially considering the *Actinomycetales* order. Such results were expected as cadmium is known to be among the most harmful heavy metal for microorganisms (Nies 1999). Decrease in pH is known to induce a decline in the bacterial structural and functional diversity (Anderson et al. 2009). In a detailed analysis of tribes within the phylum *Actinobacteria*, Newton et al. (2007) recorded distribution patterns along pH gradients revealing that lake pH is a strong predictor of the community composition in this particular phylum. Interestingly, DOC is a parameter directly related to the organic matter decomposition activity of the bacterial community. In this respect, *Actinobacteria* are believed to play a major role in organic matter degradation (Steger et al. 2007). Taken together, it suggests that the significant correlation between the low values of DOC and the drastic decrease of actinobacterial strains may be attributed to the loss of related metabolic pathways induced by heavy metal contamination.

Interphylum correlation analyses revealed that the *Actinobacteria* were shared an inverse relationship with the *Proteobacteria*. An inverse correlation was also detected between the *Bacteroidetes* and the *Proteobacteria*, despite the fact that the *Bacteroidetes* showed only marginally significant correlations with both cadmium and pH when considering the structure and diversity levels (data not shown). We can therefore conclude that there were strong patterns of co-variation between phyla, suggesting that interphylum interactions are affected in parallel across communities when impacted by a similar perturbation. This result suggests in turn that taxonomic interphylum interactions are likely conserved.

The significant correlations found with the anova analyses between environmental parameters and the phylogenic survey strongly suggest that both cadmium and pH exert the widest influence over the diversity and structure of bacterial communities. Furthermore, network analyses highlight that the pH parameter was negatively correlated with the *Acidisphaera*, meaning that acidic waters favor this particular genus, consistent with its extensive isolation from acidic environments (Hiraishi et al. 2000; Johnson et al. 2001). However, as revealed by the network analyses, cadmium is by far the parameter that exerted the widest negative impact over the abundance of the dominant species. Effectively, multiple negative correlations were found with cadmium, negatively affecting the abundance of 12% of the dominant species. Such findings strongly suggest that cadmium disturbs a significant proportion of the microbial biosphere as hosted species interact through taxonomic networks. Because dominant species have a large impact on the cycling of important nutrients (Malmstrom et al. 2004; Campbell et al. 2011), our results strongly suggest that cadmium would severely impede a variety of ecosystem functions. As a consequence, highly impacted sites have lower DOC content due to the reduced bacterial decomposition activity (Doelman and Haanstra 1979; Kelly and Tate 1998; Kelly et al. 1999). Such observations may explain that the genus *Ferrimocrobium*, although commonly found in acid mine drainages due to its capacity for ferrous iron oxidation, showed a lower abundance in the highly impacted sites where the lowest DOC values would result from weaken bacterial activities. This particular genus may therefore regroup sensitive bacterial species with great potential for assessing the intensity of a disturbance over the bacterial activities sustaining ecosystem services such as the organic matter decomposition.

Overall, our study brought evidence confirming the hypothesis that following a strong perturbation, sensitive bacterial lineages are eliminated, thus allowing opportunistic species to dominate the emptied niches. The disappearance of sensitive species gradually drives the ecosystem to an overall decrease in biodiversity, which in turn favors biotic homogenization (McKinney and Lockwood 1999). To this respect, detecting dynamic changes of both conserved taxonomic interphylum interactions and species level interaction networks provides new insights to understand further the consequences of a homogenization phenomenon that have been widely mentioned in the literature (Kandeler et al. 1996; Pennanen et al. 1996; Aoyama and Nagumo 1997; Giller et al. 1998; Kelly et al. 1999).

In conclusion, our results demonstrate that heavy metal contamination caused significant shifts within lacustrine bacterial communities both at the membership and structure levels, and drove parallel adaptation of two independent bacterial communities impacted by a similar selective pressure. Determining the extent to which ecologically relevant traits are phylogenetically conserved in such a parallel way is critical to understand the parameters that control the community phylogenetic structure, and their evolutionary consequences. In this respect, the parallel changes of interacting taxonomical networks would suggest that HGT of heavy metal resistance genes were limited to closely related species, as argued by Andam and Gogarten (2011). Otherwise, different patterns of interacting taxa would have been observed among independent contaminated lake communities. Therefore, investigating the functional repertories that were favored (gained) or disfavored (loss) in contaminated lakes will allow assessing further whether the taxonomic diversity of bacterioplankton communities primarily influenced the pathways of metabolic response to different environmental factors, as suggested by Comte and del Giorgio (2011). This study is underway.

Finally, cadmium and acidic pH were identified as the physicochemical parameters that exerted the strongest influence on the whole community taxonomic network. These two physicochemical parameters favored the *Alphaproteobacteria*, which were observed to have a functional repertory enriched in detoxifying genes (Debroas et al. 2009). Consequently, with respect to pollution control, this phylum may regroup species with valuable properties with regard to (i) hastening the mobilization of metals (with a possible application in biomining) (Valls and de Lorenzo 2002), (ii) designing metal-tolerant strains that are better adapted to perform biodegradation of organic pollutants, and (iii) metal bioremediation or mitigation through the breeding of natural or engineered strains.

Such insights therefore clearly demonstrate the need to study bacterial community diversity and structural changes triggered by abiotic contaminant exposure from an ecological and evolutionary point of view. Heavy metals affect the bacterial communities that occupy different ecological niches and participate in a variety of biogeochemical processes and ecosystem functions. As the world's freshwater resources are under severe stress, next-generation sequencing and bioinformatics are promising avenues to explore the functional interacting network adaptability of bacterial communities, and in turn, predict their capacity to maintain ecosystem homeostasis when facing rapid environmental changes.

## Data archiving statement

Data for this study are available at the NCBI Sequence Read Archive (http://trace.ncbi.nlm.nih.gov/Traces/sra/).
